# Flexible Neural Probes with Optical Artifact-Suppressing Modification and Biofriendly Polypeptide Coating

**DOI:** 10.3390/mi13020199

**Published:** 2022-01-27

**Authors:** Minghao Wang, Ye Fan, Lili Li, Fei Wen, Bangbang Guo, Minyi Jin, Jiahui Xu, Yuhao Zhou, Xiaoyang Kang, Bowen Ji, Yuhua Cheng, Gaofeng Wang

**Affiliations:** 1 Wenzhou Institute of Hangzhou Dianzi University, Wenzhou 310012, China; mhwang@hdu.edu.cn (M.W.); 16041701@hdu.edu.cn (Y.F.); 2 MOE Engineering Research Center of Smart Microsensors and Microsystems, School of Electronics & Information, Hangzhou Dianzi University, Hangzhou 311101, China; lilili@hdu.edu.cn (L.L.); wenfei@hdu.edu.cn (F.W.); bbguo@hdu.edu.cn (B.G.); Jinmy@hdu.edu.cn (M.J.); 212040242@hdu.edu.cn (J.X.); 3 The Unmanned System Research Institute, Northwestern Polytechnical University, Xi’an 710072, China; zyh160@mail.nwpu.edu.cn; 4 Collaborative Innovation Center of Northwestern Polytechnical University, Shanghai 201108, China; 5 Laboratory for Neural Interface and Brain Computer Interface, Institute of AI and Robotics, Academy for Engineering and Technology, Fudan University, 220 Handan Rd., Yangpu District, Shanghai 200433, China; xiaoyang_kang@fudan.edu.cn

**Keywords:** neural probe, electrochemical modification, photoelectric noise, neural interface, polypeptide

## Abstract

The advent of optogenetics provides a well-targeted tool to manipulate neurons because of its high time resolution and cell-type specificity. Recently, closed-loop neural manipulation techniques consisting of optical stimulation and electrical recording have been widely used. However, metal microelectrodes exposed to light radiation could generate photoelectric noise, thus causing loss or distortion of neural signal in recording channels. Meanwhile, the biocompatibility of neural probes remains to be improved. Here, five kinds of neural interface materials are deposited on flexible polyimide-based neural probes and illuminated with a series of blue laser pulses to study their electrochemical performance and photoelectric noises for single-unit recording. The results show that the modifications can not only improve the electrochemical performance, but can also reduce the photoelectric artifacts. In particular, the double-layer composite consisting of platinum-black and conductive polymer has the best comprehensive performance. Thus, a layer of polypeptide is deposited on the entire surface of the double-layer modified neural probes to further improve their biocompatibility. The results show that the biocompatible polypeptide coating has little effect on the electrochemical performance of the neural probe, and it may serve as a drug carrier due to its special micromorphology.

## 1. Introduction

Neural microelectrodes can not only record the electrical signal of neurons, but can also affect the activity of neurons and treat many diseases through stimulation. However, the small electrode size of the neural probe leads to an increase in impedance, which results in a significant reduction in the quality of single-unit recording. Therefore, some novel neural interface materials have been explored to realize low impedance and high signal quality without compromising spatial resolution. Poly(3,4-ethylenedioxythiophene) (PEDOT) is a conductive polymer that has been utilized to improve electrochemical stability of neural microelectrode [1,2,3,4,5,6,7,8]. PEDOT deposition on microelectrodes can decrease impedance and improve charge injection limit significantly. Moreover, water-soluble poly(styrenesulfonate) (PSS) can be adopted as a counter-ion of PEDOT to electrodeposit stable conductive polymer in aqueous solution. Therefore, PEDOT-PSS is widely used due to its good stability, excellent electronic conductivity and easy processability [9,10,11,12,13,14]. Recently, graphene oxide (GO) has also been doped into PEDOT to obtain multifunction due to its excellent electrochemical and mechanical properties [15,16,17]. Therefore, it is necessary to compare the effectiveness of various PEDOT-based materials in order to improveme electrochemical performance and mechanical stability of the neural interface.

On the other hand, a neural optrode that can perform both optical stimulation and electrical recording has been developed and widely used in neuroscience research. Due to the existence of high-magnitude optical stimulation artifacts in the recorded signal, the optrode cannot provide both stimulating and recording capacities at high spatiotemporal resolutions [18]. Meanwhile, the optical stimulation has to be limited to a slowly changing low-frequency signal, or the signals, which were collected within a few milliseconds around the beginning and end of the pulsed optical simulation, had to be discarded from the recorded neural signal [19,20]. The optical stimulation artifacts mainly come from electromagnetic interference (EMI), photoelectrochemical effect (PEC) and photovoltaic effect (PV). Recently, the EMI noise (up to 5.8 mVpp) has been suppressed by applying metal shielding layers and using transient pulse shaping of LED drive signals [21]. Meanwhile, the PV noise (up to 1 mVpp) can be mitigated by using heavily boron-doped silicon substrate or eliminated by using flexible polymer-based substrates [22,23]. Furthermore, the PEC noise (up to 200 µVpp) on the metal–electrolyte interface can be mitigated by using electrochemical modification of materials with a band gap of larger than 3.26 eV, such as counterion-doped PEDOT, Sn-doped indium oxide or graphene [24,25]. In a previously published work [26], it was demonstrated that graphene oxide-doped PEDOT (PEDOT-GO) can effectively reduce PEC noise. However, direct comparison of electrochemical performance, stability and PEC noise of the PEDOT-GO modifications with the PEDOT-PSS modifications has not been reported.

Research on implantable microelectrodes demonstrated that, except for reducing implantation footprint [27] and mechanical modulus [28], the coating of biocompatible material at the microelectrode surface can determine the level of inflammatory reaction and gliotic encapsulation near neural implants. Examples of these biocompatibility maintenance methods [29] include controlled release of curcumin [30], dexamethasone [31,32] and other anti-inflammatory agents [33], functionalization with neuron adhesion factors [34], and hydrogel coatings [35]. Furthermore, it was demonstrated that silk coatings may reduce some markers of gliosis in an in vitro model and that silk can encapsulate and release the gliosis-modifying enzyme chondroitinase ABC [36].

In this work, a flexible polyimide (PI)-based neural probe is fabricated by micromachining technology to serve as the test object without PV noise. Then, five kinds of neural interface materials are deposited on the PI-based probes and illuminated with a series of blue laser pulses to compare their electrochemical performance, stability and photoelectric artifact. Firstly, electrochemical characteristics of the modified microelectrodes are obtained by measuring charge storage capacity (CSC) and electrochemical impedance spectra (EIS). Secondly, mechanical stability of the modified microelectrodes is studied by comparing electrochemical characteristics (by CSC and EIS) and microstructures (by SEM) before and after cyclic voltammetry (CV) scanning. Thirdly, photoelectric artifacts of the modified microelectrodes are analyzed by measuring background noise and PEC noise separately. Finally, a layer of polypeptides is deposited on the surface of the modified neural probes to improve their biocompatibility and realize drug delivering.

## 2. Materials and Methods

### 2.1. Reagents and Apparatus

Graphene oxide aqueous solution (1 mg/mL) was obtained from Suzhou TANFENG Graphene Technology Co., Ltd. (Suzhou, China). Phosphate buffered saline (PBS, pH 7.4) was obtained from Sinopharm Chemical Reagent Co., Ltd. (Shanghai, China). Ethylenedioxythiophene (EDOT) was purchased from Cool Chemical Technology Co., Ltd. (Beijing, China). PSS was purchased from Herochem Co., Ltd. (Shanghai, China). Phenylalanine-phenylalanine (Phe-Phe) was produced by GL Biochem Co., Ltd. (Shanghai, China). Laser diodes (445 nm, 80 mW) were acquired by SINOSEMIC Co., Ltd. (Jinan, China). Anisotropic conductive films (ACF) were produced by HITACHI, Ltd. (Tokyo, Japan). Glucose levels were measured using a home glucose meter (ONETOUCH Veriovue, Johnson & Johnson, New Brunswick, NJ, USA). Scanning electron microscopy (SEM) was analyzed by a high-vacuum scanning electron microscopy (Zeiss Sigma 300, Oberkohen, Germany). The electrochemical measurements and modification were carried out by an electrochemical workstation (CHI660E, CH instrument, Shanghai, China). Noise signals were collected by an RHD2000 Evaluation System (Intan Technology, Los Angeles, CA, USA).

### 2.2. Flexible Probes Fabrication

**Figure 1:**
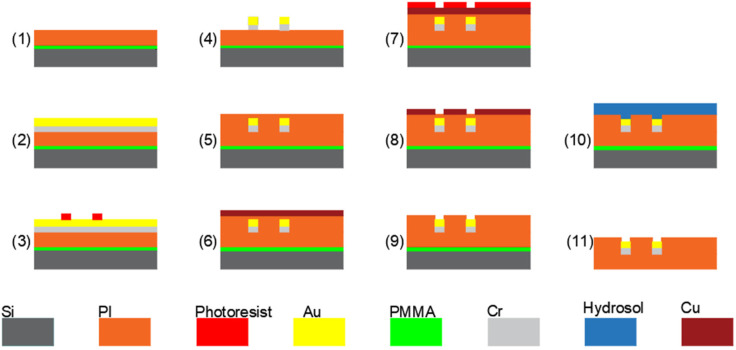
Fabrication process of the flexible PI probes.

**Figure 2:**
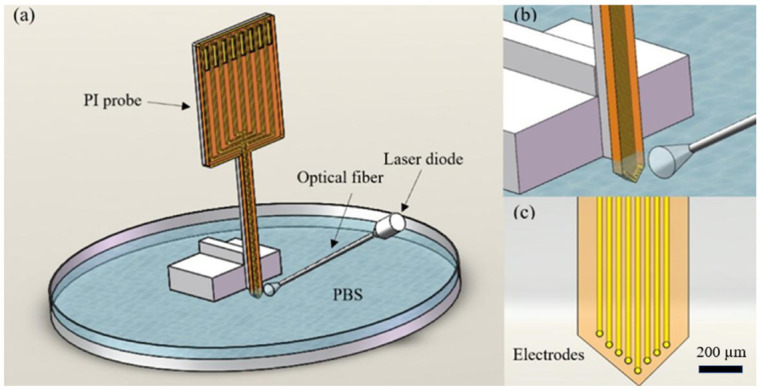
Schematic diagram of (**a**) assembled bench noise measurement system and (**b**,**c**) local enlarged drawing of eight microelectrodes with diameter of 25 μm and pitch of 70 μm.

The microfabrication process of the PI-based probes is depicted in Figure 1. Firstly, a single-side oxidized and polished P-type silicon wafer is used as the substrate. The thickness of the oxide and the silicon of the wafer are 0.2 µm and 400 µm, respectively. Secondly, a layer of polymethyl methacrylate (PMMA) is spin-coated on the wafer as a sacrificial layer and 50 µm-thick commercial PI tape (3M China Co., Ltd., Shenzhen, China) is attached to the wafer using a film laminator to serve as the lower dielectric layer. Thirdly, Cr/Au layers with thickness of 20/200 nm are sputtered on PI to serve as the conductive layer. The conductive layer is then patterned by photoresist and etched in Cr/Au etching solution to form the recording microelectrodes, conductive pads and traces. Next, a 5 um-thick PI layer (PAA-1002, Changzhou ya’an new material Co. LTD, Changzhou, China) is spin-coated on the wafer to serve as the top dielectric layer. Then, another 100 nm-thick Cu layer is sputtered and patterned by photoresist to serve as mask, and reactive ion etching (RIE) is conducted to expose the microelectrode sites and bonding pads. The device is gently peeled off from the silicon wafer by the hydrogel transfer method. As illustrated in Figure 2, the optrode has one shank with dimensions of 5 mm in length, 500 μm in width and 55 μm in thickness, where eight microelectrodes are arranged along the tip of the shank in an arrow shape with diameter of 25 μm and pitch of 70 μm.

### 2.3. Electrochemical Modification

To compare photoelectric noises and electrochemical properties of the modified PI probes, five identical devices each with eight microelectrodes are electrodeposited with Pt-Black (PBK), PEDOT-PSS (PPS), PEDOT-GO (PGO), Pt-Black/PEDOT-PSS (PBK/PPS) and Pt-Black/PEDOT-GO (PBK/PGO), separately. In detail, PBK is electrodeposited by applying repeated current pulses (peak current density of 4.5 A/cm^2^, duty ratio of 5 ms: 495 ms, cycles of 200) in chloroplatinic acid solution (3% chloroplatinic acid and 0.01% lead acetate in deionized water). For PPS deposition, 250 mg PSS is dissolved in 50 mL of deionized water. Then, 0.01 M EDOT is dropped in the solution and stirred for 1 h to produce the electrolyte. The PPS is electrodeposited by applying a constant current with current density of 0.2 mA/cm^2^ and deposition time of 10 min. For PGO deposition, GO aqueous solution (1 mg/mL) is ultrasonicated for 30 min to disperse it evenly. Then, 0.01 M EDOT is dropped in the solution and stirred for 1 h to obtain the electrolyte. The PGO is electrodeposited by applying a constant current with current density of 0.2 mA/cm^2^ and deposition time of 10 min.

### 2.4. Bench Noise Measurement

The schematic diagram of the bench noise measurement system is described in Figure 2. The system includes a flexible PI probe, a laser diode (LD)-coupled optical fiber and an electrolytic cell. In order to achieve signal recording, the PI probe is bonded to the flexible conductive cable by anisotropic conductive adhesive (ACF)-bonding. Then, the bonded flexible PI probe is fixed on a glass slide and immersed in PBS solution to test photoelectric artifact in a foil-shielded box. An LD-coupled multimode glass fiber (0.22 NA) with a core/cladding diameter of 105/260 μm is used as a light source to expose the microelectrodes to light illumination directly. The vertical distance between the optical fiber tip and the plane where the microelectrode points are located is fixed at 1 cm, making the power of the light illuminated on the microelectrodes remain consistent. The noise signals are recorded by the RHD2000 Evaluation System. The cut-off frequency of the first order Butterworth high-pass filter is 250 Hz, and the data sampling frequency is 20 kHz. A series of blue light pulses (450 nm, 5 ms) are applied to research the photoelectric noise. The amplitude of photoelectric noise is defined as the generated absolute peak-to-peak voltage amplitude during light on.

### 2.5. Biocompatible Polypeptide Coating

The biocompatible polypeptide coating is deposited from a Physical Vapor Deposition (PVD) system (SKY RH-300, SKY Technology Development Co., Ltd., Shenyang, China). For this purpose, 10 mg of Phe-Phe polypeptide lyophilized powder is put into the evaporation chamber of the PVD system, and the flexible probe is put into the deposition chamber of the PVD system. After the deposition chamber is vacuumed to less than 10^−3^ Pa, the temperature of the evaporation chamber is raised to 220 °C to achieve polypeptide deposition. The deposition time is about 10 min, during which the polypeptides in the evaporation chamber can evaporate completely.

## 3. Results

### 3.1. Electrochemical Characterization

**Figure 3:**
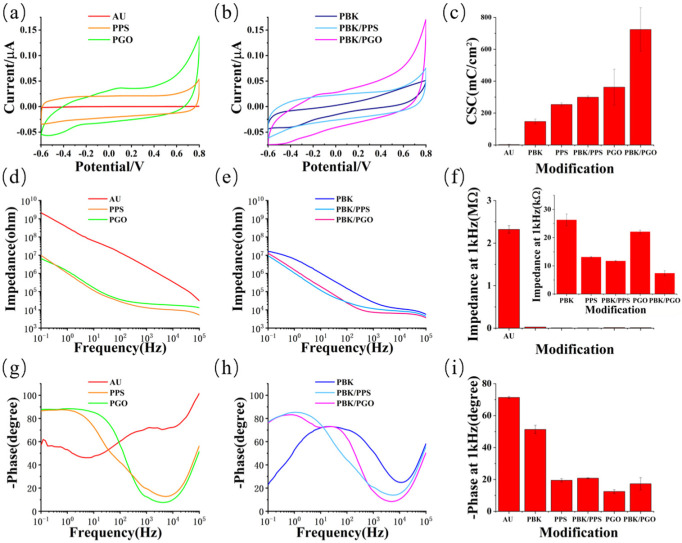
Electrochemical characterization of unmodified, PBK-modified, PPS-modified, PBK/PPS-modified, PGO-modified, and PBK/PGO-modified probes. (**a**,**b**) Mean (±SD) cyclic voltammetry curves and (**c**) corresponding CSC Values; (**d**,**e**) mean electrochemical impedance spectra and (**f**) corresponding impedance at 1 kHz; (**g**,**h**) mean phase curves and (**i**) corresponding phase delay at 1 kHz (*n* = 3).

In order to compare electrochemical properties of the modified microelectrodes, the CV and EIS are tested in PBS with a saturated calomel electrode (SCE) as reference electrode and a Pt foil as counter electrode. The EIS is tested at a frequency range from 10^−1^ Hz to 10^5^ Hz with input voltage amplitude of 10 mV, and the CV is scanned over the potential range between −0.6 V and 0.8 V at the scanning rate of 0.1 V/s. The CV and EIS from three microelectrodes of the PI probe are tested and calculated with averages for the bare Au microelectrode and the five modified microelectrodes. The enclosed area of the CV curve represents the CSC of the microelectrodes with different modifications. By comparison, the CSC of the bare gold microelectrodes significantly increases after modification. As depicted in Figure 3b, the CSC of the PBK/PPS-modified microelectrode is much larger than that of the PPS-modified ones. Meanwhile, the CSC of the PBK/PGO-modified microelectrode is significantly larger than that of the PGO directly modified ones. In general, the PBK/PGO-modified microelectrodes exhibit the largest enclosed area of the CV curves. As illustrated in Figure 3c,d, the impedance of the bare gold microelectrodes significantly decreases at 1 kHz after modification. The averaged impedance and phase delay at 1 kHz of the unmodified, PBK-modified, PPS-modified, PBK/PPS-modified, PGO-modified, and PBK/PGO-modified microelectrodes are listed in Table 1. Results show that the microelectrodes modified with PBK/PGO and PBK/PPS both have lower impedance at 1 kHz than those modified with PGO and PPS directly. Owing to the slight diffusion of PBK/PGO, the geometric surface area of the PBK/PGO-modified microelectrode is actually larger than that of the PBK/PPS-modified one. Thus, the effect of the PBK/PGO modification and the PBK/PPS modification on the improvement of CSC and reduction in impedance and phase delay of the microelectrode may be similar.

**Figure 4:**
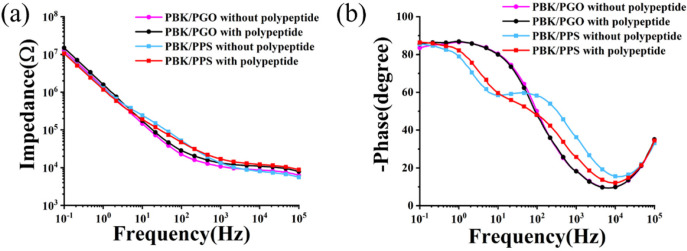
Mean (±SD) (**a**) EIS and (**b**) phase plots of PBK/PGO- and PBK/PSS-modified probes with or without polypeptide coating (*n* = 3).

In order to examine the effect of polypeptide coating on the electrochemical property of the flexible probes, the AC impedance spectra before and after polypeptide deposition are measured and compared in Figure 4a,b. As illustrated in Table 1, the impedance and phase delay of the PBK/PGO- and PBK/PSS-modified microelectrodes are not much affected by the polypeptide covering on the microelectrodes. This may be due to the loose and porous surface structure of polypeptides, which is ideal for ion transport and charge transfer. These porous structures serve as conductive channels of the microelectrodes, thus ensuring excellent transmission of neural signals.

### 3.2. Stability Characterization

**Figure 5:**
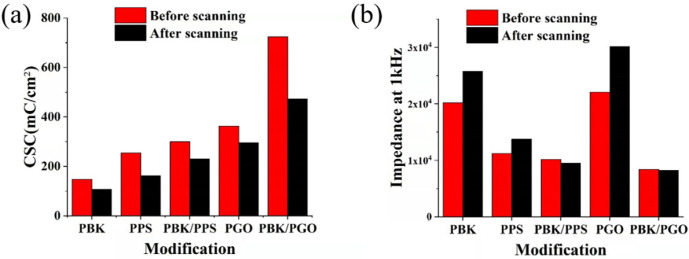
(**a**) CSC plots and (**b**) impedance at 1 kHz before and after 2000 cycles of CV scanning.

Previous studies have shown that PBK, PPS and PGO all have good biocompatibility [37,38]. These materials may not be biotoxic when implanted into the body, while the delamination of the modification materials from the microelectrode will affect its electrochemical characteristics [39]. Therefore, mechanical stability of the modified microelectrodes is tested by CV scanning. The CV scanning is performed between −0.6 V and 0.8 V in PBS solution with a scanning rate of 1 V/s. After scanning, the CV and EIS are measured and the corresponding CSC and impedance are recorded and depicted in Figure 5a,b. The results show that the CSCs of the modified microelectrodes all reduce to a certain degree after 2000 cycles of scanning. The decrease in CSC may be caused by the partial shedding of the modification materials from the microelectrodes. It is worth noting that the variation trend of the impedance at 1 kHz during the scanning is not the same. In comparison, the impedance of PBK, PPS and PGO shows an obvious increase (27.7%, 22.3% and 36.5%) in magnitude, while the impedance of PBK/PGO and PBK/PPS shows a small decrease (−6.6% and −1.9%) in magnitude. In addition, the impedance variation amplitudes of the PBK/PPS- and PBK/PGO-modified microelectrodes are also less than those of the PPS- and PGO-modified ones. As the impedance variation can reflect the adhesion strength between the materials and the microelectrodes, it can be concluded that the adhesion strength of the double-layer PBK/PPS and PBK/PGO is greater than that of the single-layer PBK, PPS and PGO. The reason may be that the rough surface of PBK can increase the adhesion force between the microelectrode and the PPS/PGO, which makes the PBK/PPS and PBK/PGO more stable. The CV scanning demonstrates that the PBK/PGO- and PBK/PPS-modified microelectrodes have the best electrochemical stability under electrical stimulation or recording. When PPS/PGO is used for electrochemical modification, it is better to electroplate PBK before electroplating PPS/PGO on the gold microelectrode than to electroplate PPS/PGO directly on the gold microelectrode.

### 3.3. Morphology Characterization

**Figure 6:**
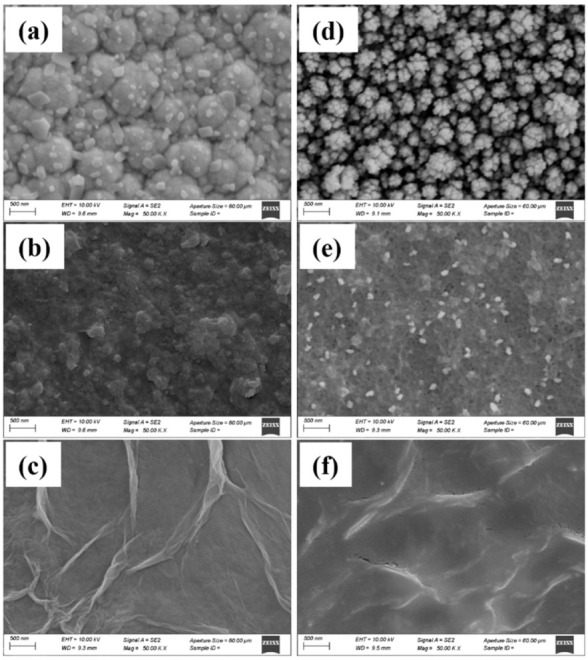
SEM images of (**a**) PBK-, (**b**) PBK/PPS-, and (**c**) PBK/PGO-modified microelectrode before CV scanning. Corresponding SEM images of (**d**) PBK-, (**e**) PBK/PPS- and (**f**) PBK/PGO-modified microelectrode after 2000-cycle CV scanning.

The surface morphology of microelectrode sites, especially the specific surface area, directly determines the charge transfer and diffusion process between the electrode–electrolyte interface. Therefore, the properties of electrochemical impedance and phase delay are significantly influenced by the morphology of the neural interface materials. The SEM pictures of the PBK, PKB/PPS and PBK/PGO are depicted in Figure 6. As shown in Figure 6a, the as-deposited PBK is composed of many cauliflower-shape particles that have a large effective surface area and are tightly bound to the gold substrate. Figure 6b shows the SEM pictures of another PBK-modified microelectrode after 2000-cycle CV scanning. Although this PBK-modified microelectrode appears to be thinner, no significant cracking is observed. The SEM pictures in Figure 6c show that the as-deposited PBK/PPS exhibits a relatively even surface with small island dimensions. The interconnections between islands are established to form a porous frame in the nanometer scale. After the CV scanning, the surface morphology of PBK/PPS appears more microporous, as shown in Figure 6d. These micropores can accelerate the charge transfer rate so that the impedance at 1 kHz of PBK/PPS decreases rather than increases after the CV scanning. The SEM picture in Figure 6e shows that the as-deposited PBK/PGO leads to a fold structure with large specific surface area, which helps to improve the electrochemical performance. After the CV scanning, the morphonology of PBK/PGO appears to have more cracks between the folds, as shown in Figure 6f. The cracks can also accelerate the charge transfer rate and make the impedance at 1 kHz of PBK/PGO decrease rather than increase after the CV scanning. The SEM pictures reveal that the variation in CSC and impedance is due to the morphology change during the CV scanning. The film undergoes reduction and oxidation with corresponding movement of ions in or out the film. In this reversible motion, the ions cause the film to expand and contract repeatedly, and eventually make the film crack, delaminate and perforate. On the one hand, film delamination can lead to a decrease in CSC and an increase in impedance. On the other hand, perforation and cracks may in turn increase CSC and decrease impedance. Therefore, PBK/PPS and PBK/PGO may have a lower rate of film delamination and a higher rate of perforation and cracks during scanning than PPS and PGO, which makes their impedance drop rather than rise.

**Figure 7:**
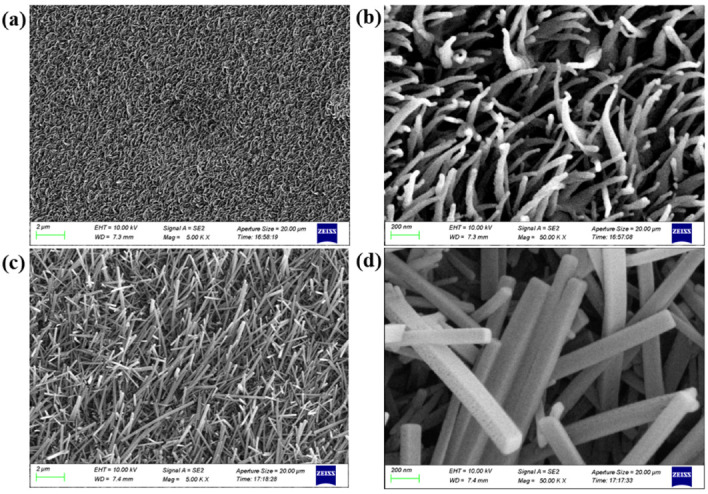
(**a**,**b**) SEM images of the as-deposited polypeptide on PI probes. (**c**,**d**) SEM images of the polypeptide after being immersed in PBS solution for 10–15 min.

The polypeptide deposited from the PVD system covers the entire surface of the flexible probe, and the surface morphology of the microelectrode sites is changed. The morphology of the polypeptide-coated neural probes is characterized and depicted in Figure 7. As shown in Figure 7a,b, the as-deposited polypeptide is composed of many fluffy nanofibers that have a diameter in the tens of nanometers and a thickness of 3.5 micrometers. Most of these nanofibers are curved in shape and arranged haphazardly. As polypeptides may degrade due to exposure to PBS or bodily fluids during in vitro testing and in vivo implantation, the morphology of polypeptide soaked in PBS is also characterized. The SEM images of the polypeptide after being immersed in PBS solution for 10–15 min are shown in Figure 7c,d. It is clear that the nanofibers become thicker and straighter after soaking, and they all have a prismatic structure with a diameter of 100–200 nm. The change in morphology after soaking is caused by water absorption and swelling of the polypeptide in PBS.

### 3.4. Bench Noise Tests

**Figure 8:**
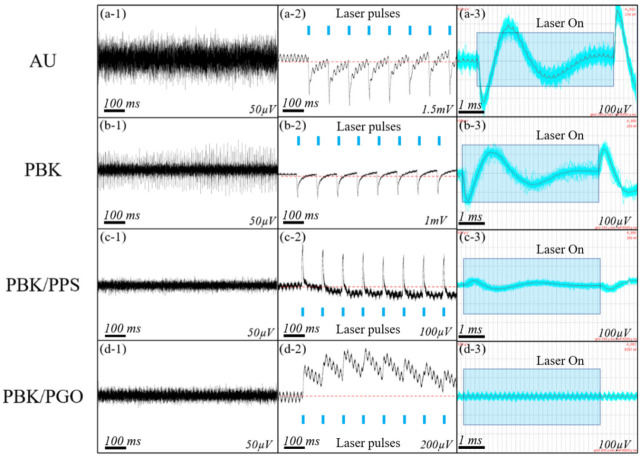
Background noise recorded from (**a-1**) gold microelectrode, (**b-1**) PBK-modified microelectrode, (**c-1**) PBK/PPS-modified microelectrode and (**d-1**) PBK/PGO-modified microelectrode on bench (50 µV represents half of the full vertical scale). (**a-2**–**d-2**) Recorded full-pass PEC noise of PI-based probe under laser pulse stimulation (0.2 A, 5 ms, 10 Hz). (**a-3**–**d-3**) Recorded high-pass PEC noise of PI-based probe under laser pulse stimulation (0.2 A, 5 ms, 10 Hz, *n* = 100).

The waveforms of photoelectric noise and background noise recorded in PBS solution are depicted in Figure 8. As shown in Figure 8(a-1–d-1), the background noises of the unmodified, PBK-modified, PBK/PPS-modified and PBK/PGO-modified microelectrodes are 42 ± 2.9 µV, 30 ± 1.7 µV, 15 ± 0.6 µV and 12 ±0.5 µV, respectively. It is obvious that the amplitude of the background noise is positively correlated with the impedance of the modified microelectrode. As can be seen in Figure 8(a-2–d-2), the all-pass PEC noise amplitude of the bare gold microelectrode decreases significantly after PBK, PBK/PPS and PBK/PGO modifications. In particular, the all-pass PEC artifacts of the gold and PBK-modified microelectrodes from the PI-based optrode all have a “v”-shaped waveform at the onset of the optical stimulation. However, the polarity of the PEC artifacts of the PBK/PPS-modified and PBK/PGO-modified microelectrodes is inverted, making an “inverted-v” (or “Λ”)-shaped waveform. This phenomenon is because the PEC of Au/PBK (metal) leads to the accumulation of electrons, whereas the PEC of the microelectrode modified by PPS/PGO (conductive polymer) leads to the accumulation of holes. Therefore, for PBK/PPS and PBK/PGO, the polarization direction of the PEC noise from their modified microelectrodes is determined by the sum of the PEC noise of PBK, PPS and PGO. As shown in Figure 8(a-3–d-3), the average amplitude of high-pass PEC artifacts of the unmodified and PBK-modified microelectrodes is 141 ± 23.68 µV and 87 ± 14.51 µV, respectively. After being modified with PBK/PPS and PBK/PGO, the average amplitude of the high-pass PEC noise is barely visible in the background noise. The results show that the high-pass PEC noise amplitude of the bare gold microelectrode also decreases significantly after PBK/PPS and PBK/PGO modifications. In general, the PBK/PPS-modified microelectrode has the lowest all-pass PEC noise amplitude, while after bandpass filtering, the PBK/PGO-modified microelectrode has the lowest high-pass PEC noise amplitude. Considering the electrochemical performance, stability, background noise and PEC noise, the double-layer PBK/PPS and PBK/PGO may be the best modification materials for the neural interface.

## 4. Conclusions

In this work, five kinds of neural interface materials were deposited on PI-based microelectrodes to compare their electrochemical performance, stability and photoelectric artifact. Firstly, the morphology characterization showed that the PBK/PGO and PBK/PSS were deposited on microelectrodes evenly and tightly without diffusion and delamination. Secondly, the electrochemical characterization illustrated that the microelectrode modified with PBK/PGO and PBK/PPS both have larger CSC and lower impedance at 1 kHz than those modified with PGO and PPS directly. Thirdly, the stability characterization demonstrated that the PBK/PGO- and PBK/PPS-modified microelectrodes have the best electrochemical stability during CV scanning. Lastly, the bench noise recordings revealed that the PBK/PPS-modified microelectrode has the lower all-pass PEC noise amplitude, while the PBK/PGO-modified microelectrode has the lower high-pass PEC noise amplitude. After comprehensive consideration of the electrochemical performance, stability and bench noise, the double-layer PBK/PPS or PBK/PGO may be the best candidates for high-stability and low-artifact optogenetics applications. It is worth mentioning that the biocompatible polypeptide coating has little effect on the electrochemical performance of the neural probe, and it may serve as a drug carrier due to its special micromorphology. However, the SEM results suggested that the polypeptide would absorb water and swell in PBS and eventually degrade. Therefore, how to slow down the degradation rate of polypeptides to achieve long-term biocompatibility maintenance in vivo is the next problem that needs to be solved. In addition, the in vitro cytotoxicity and in vivo biocompatibility of polypeptides also need to be further verified.

## Figures and Tables

**Table 1:** The mean (±SD) impedance and phase delay at 1 kHz of the unmodified, Pt-Black (PBK), PEDOT-PSS (PPS), PBK/PPS, PEDOT-GO (PGO), PBK/PGO, PBK/PPS-with-polypeptide- and PBK/PGO-with-polypeptide-modified microelectrodes (*n* = 3).

Item	AU	PBK	PPS	PBK/PPS	PGO	PBK/PGO	PBK/PPS with Polypeptide	PBK/PGO with Polypeptide
Impedance (kΩ)	2330.0 ± 89.2	26.3 ± 2.0	13.1 ± 0.2	11.7 ± 0.2	22.1± 0.6	7.4 ± 0.9	16.9 ± 1.5	13.4 ± 0.9
Phase delay (degree)	71.4 ± 0.6°	51.3 ± 2.5°	19.5 ± 1.0°	20.8 ± 0.2°	12.5 ± 1.1°	17.3 ± 3.8°	25.7 ± 1.5°	18.2 ± 0.8°
